# A Unique Presentation of Nelson Syndrome Due to Partial Adrenal Insufficiency Without Bilateral Adrenalectomy

**DOI:** 10.7759/cureus.43518

**Published:** 2023-08-15

**Authors:** Priyanka D Desai, Smita Kargutkar, Raveena Nalla

**Affiliations:** 1 Biomedical Engineering, Cornell University, Ithaca, USA; 2 Endocrinology, Diabetes, and Metabolism, ACE Endocrinology Associates, Red Bank, USA; 3 Endocrinology, Diabetes, and Metabolism, Monmouth Medical Center, Long Branch, USA

**Keywords:** acth-secreting macroadenoma, low am cortisol, high acth, skin hyperpigmentation, nelson syndrome

## Abstract

Nelson syndrome is a rare disorder, characterized by clinical features arising from an adrenocorticotropic hormone (ACTH)-secreting pituitary macroadenoma after bilateral adrenalectomy. Common symptoms of Nelson syndrome include weight gain, vision problems, and skin hyperpigmentation, among many others. In this case report, a 58-year-old Asian female who displayed clinical features akin to Nelson syndrome despite not undergoing bilateral adrenalectomy is investigated. The patient has a past history of an ACTH-secreting pituitary macroadenoma, for which a transsphenoidal resection was performed along with radiation therapy. A year following this, she displayed severe facial and neck hyperpigmentation.

According to the laboratory results obtained, the patient displayed initial high ACTH levels and low-normal AM cortisol levels, which are signs of partial adrenal insufficiency. A brain MRI was performed, which confirmed stable residual tumor tissue in the cavernous sinus. The results pointed to the adrenal glands as the cause of the hyperpigmentation, and the patient was diagnosed with primary adrenal insufficiency.

To bring her ACTH levels and low-normal AM cortisol into the proper range, she was given low-dose hydrocortisone and monitored for five years. Over this time period, her hyperpigmentation improved significantly and eventually resolved entirely, and her ACTH levels were lowered, indicating that hydrocortisone was the appropriate treatment for normalizing ACTH levels. In this case, it was determined that unresponsive adrenal glands lead to high ACTH levels, which resulted in an atypical case of Nelson syndrome and the physical symptom of hyperpigmentation.

## Introduction

Nelson syndrome refers to a spectrum of clinical features arising from an adrenocorticotropic hormone (ACTH)-secreting pituitary macroadenoma after therapeutic bilateral adrenalectomy [1]. Nelson syndrome is a rare disorder that occurs in about 8%-47% of patients who have undergone bilateral adrenalectomy for Cushing disease [2]. The clinical signs and symptoms observed relate to the local effects of the enlarging pituitary adenoma on the surrounding structures, excess adrenocorticotropin secretion [3], hyperpigmentation of the skin from high ACTH levels, and secondary loss of other pituitary hormones [1]. More than 99% of cases of Nelson syndrome follow bilateral adrenalectomy in patients who have Cushing disease due to an ACTH-secreting pituitary adenoma. Generally, these cases are managed by surgery, radiation to the pituitary, or medically with cabergoline, octreotide, or cyproheptadine [4].

In this case, we investigate a 58-year-old Asian female with facial hyperpigmentation and ACTH-secreting pituitary adenoma who displays clinical features similar to Nelson syndrome due to partial adrenal insufficiency, without undergoing a bilateral adrenalectomy.

This article was previously presented at the American Association of Clinical Endocrinology (AACE) 28th Annual Scientific and Clinical Congress, on April 24-28, 2019, at Los Angeles Convention Center as an electronic poster.

## Case presentation

A 58-year-old Asian female presented to an outpatient endocrinology clinic with severe facial and neck hyperpigmentation. She had a history of ACTH-secreting pituitary macroadenoma, for which a transsphenoidal resection was performed eight years ago. It was observed that she had residual tumor tissue that extended into the right cavernous sinus, which she received two sessions of radiation to treat postoperatively. After microscopically examining the specimens with an immunostain profile, positive ACTH and focally positive prolactin results were revealed.

Approximately one year after the surgery, she began to notice the initial signs of hyperpigmentation on her face and neck, which progressively worsened over the next eight years. After consulting multiple dermatologists, she was prescribed various topical steroid creams that had no effect in improving her hyperpigmentation. Other than the visible hyperpigmentation, the patient had no other symptoms typical of adrenal insufficiency, for example, nausea, vomiting, abdominal pain, diarrhea, myalgias, arthralgias, and weight loss.

Her physical examination was unremarkable besides the severe facial and neck hyperpigmentation. Her electrolytes were within the normal range as well. Although most of her laboratory data was within normal limits, it revealed high levels of ACTH and low-normal levels of AM cortisol (Table 1).

**Table 1:** Laboratory Investigations of Hormone and Chemical Levels ACTH: adrenocorticotropic hormone, TSH: thyroid-stimulating hormone, T4: thyroxine

Date	ACTH (normal range: 7.2-63.3 pg/mL)	AM cortisol (normal range: 6.2-19.4 ug/dL)	TSH (normal range: 0.450-4.500 uIU/mL)	Free T4 (normal range: 0.82-1.77 ng/dL)
06/05/2017	103.6 pg/mL	13.3 ug/dL	0.857 uIU/mL	1.04 ng/dL
11/03/2017	73.3 pg/mL	12.9 ug/dL	0.020 uIU/mL	1.70 ng/dL
03/23/2018	49.6 pg/mL	9.6 ug/dL	15.050 uIU/mL	0.57 ng/dL
05/18/2020	40.1 pg/mL	7.7 ug/dL	0.989 uIU/mL	1.04 ng/dL
10/31/2020	34.3 pg/mL	9.6 ug/dL	6.390 uIU/mL	0.97 ng/dL
04/28/2021	31.7 pg/mL	7.9 ug/dL	0.083 uIU/mL	1.79 ng/dL
03/31/2022	56.2 pg/mL	7.8 ug/dL	0.720 uIU/mL	1.36 ng/dL
09/21/2022	50.3 pg/mL	10.2 ug/dL	0.127 uIU/mL	1.30 ng/dL

**Figure 1:**
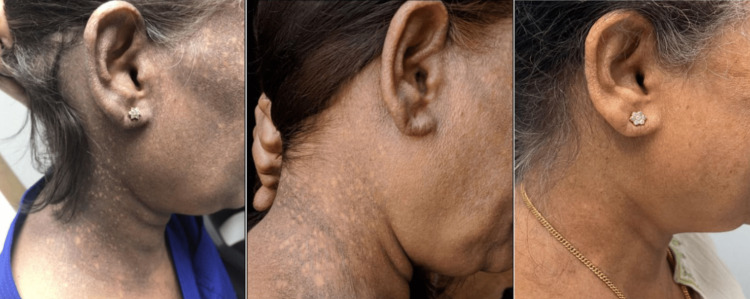
Skin Hyperpigmentation Before, During, and After Treatment With Hydrocortisone

To check for other abnormalities, investigations were performed on the brain. Performing an MRI on the brain is the standard follow-up procedure if a tumor is not detected [5]. The MRI results showed stable post-surgical changes with stable residual enhancing soft tissue along the right aspect of the sella extending into the cavernous sinus. From this, it was determined that the proper course of action would be focusing on bringing the ACTH and AM cortisol levels into normal ranges. She was then started on hydrocortisone, with a 10 mg dose in the morning and a 5 mg dose in the evening. After carefully monitoring her for the course of five years, it was observed that her hyperpigmentation improved significantly with each encounter and, in the present, has completely resolved, with visible results displayed in Figure 1. Her ACTH level was brought into the normal reference range after taking the hydrocortisone, as displayed after the dates of 03/23/2018 in Table 1.

It is imperative that other diagnoses should be considered in patients with hyperpigmentation who have not undergone adrenalectomy. In our patient, the use of low doses of hydrocortisone over the span of multiple years was effective in decreasing ACTH levels and improving hyperpigmentation significantly. This suggests that primary adrenal insufficiency was the cause of excess ACTH production.

## Discussion

The patient, who underwent transsphenoidal resection of a pituitary macroadenoma eight years prior, displayed skin hyperpigmentation on the face and neck. The reason for the sudden emergence of hyperpigmentation was unclear, and standard dermatologist-recommended creams did not reduce the discoloration. After testing the hormone levels produced by the pituitary gland, adrenal gland, and thyroid gland, it was identified that the adrenal gland was the root cause, as it was unresponsive to ACTH hormone signals and produced low levels of cortisol. Low cortisol levels in the blood led to high ACTH present, which led to the hyperpigmentation. By treating the patient with topical hydrocortisone, cortisol levels in the bloodstream were increased into a normal range, allowing the negative feedback loop to decrease ACTH levels. This significantly improved the skin hyperpigmentation.

Through this case report, it has been found that there is a relationship between high ACTH levels and skin pigmentation, and preexisting research done on primary adrenal insufficiency sheds more light on the mechanism of action. It has been found that excess production of ACTH leads to the overstimulation of melanocortin-1 receptors (MC1Rs) that reside on the surface of dermal melanocytes, and this is due to the ACTH hormone’s α-melanocyte-stimulating activity [6,7]. After stimulation, the melanocyte’s color shifts to a dark brown or black color, which was seen most visibly in the first panel of Figure 1.

The relationship between the hypothalamus, pituitary, and adrenal gland also provides clearer insight into this case study. The hypothalamus releases corticotropin-releasing hormone (CRH), which travels to the anterior pituitary gland to stimulate the production of ACTH. ACTH moves a step further into the pathway and is received by ACTH receptors of the adrenal cortex [8]. As a response to ACTH binding to receptors, the adrenal cortex secretes glucocorticoids, which triggers a negative feedback loop to alert the hypothalamus to hinder CRH production, which consequently hinders ACTH production [8]. In the case of primary adrenal insufficiency, however, glucocorticoid levels are decreased, which in turn cannot inhibit CRH and ACTH levels, causing ACTH to be produced in excess as explained previously.

## Conclusions

Primary adrenal insufficiency should be considered as a cause of excessive ACTH production resulting in hyperpigmentation and ACTH-secreting pituitary adenoma. A decrease in the responsiveness of the adrenal gland to ACTH led to a low normal AM cortisol production. Since blood cortisol did not reach a healthy level, the negative feedback loop that results to slow down ACTH production was not activated, which led to excessive ACTH levels. By using low-dose hydrocortisone to increase the blood cortisol levels to a healthy level, ACTH production was suppressed, decreasing the hyperpigmentation seen in the patient. Thus, the partial adrenal insufficiency decreased the sensitivity of the adrenal gland to ACTH, which served as the root cause of the hyperpigmentation.
